# Telemedicine treatment of patients with mental disorders during and after the first COVID-19 pandemic lockdown in Germany – an observational study on feasibility and patient satisfaction

**DOI:** 10.1186/s12888-023-05140-w

**Published:** 2023-09-05

**Authors:** Karsten Link, Svenja Christians, Wolfgang Hoffmann, Hans Jörgen Grabe, Neeltje van den Berg

**Affiliations:** 1 Institut Für Community Medicine, Universitätsmedizin Greifswald, Ellernholzstraße 1-2, 17475 Greifswald, Germany; 2 Klinik Und Poliklinik Für Psychiatrie Und Psychotherapie, Universitätsmedizin Greifswald, Ellernholzstraße 1-2, 17475 Greifswald, Germany

**Keywords:** Telemedicine, Satisfaction, Acceptance, COVID-19 pandemic, Lockdown, Telemedical treatment, Psychiatric outpatients

## Abstract

**Introduction:**

In response to the COVID-19 pandemic, a general lockdown was enacted across Germany in March 2020. As a consequence, patients with mental health conditions received limited or no treatment in day hospitals and outpatient settings. To ensure continuity of care, the necessary technological preparations were made to enable the implementation of telemedical care via telephone or video conferencing, and this option was then used as much as possible. The aim of this study was to investigate the satisfaction and acceptance with telemedical care in a heterogeneous patient group of psychiatric outpatients in Germany during the first COVID-19 lockdown.

**Methods:**

In this observational study, patients in ongoing or newly initiated outpatient psychiatric therapy as well as those who had to be discharged from the day clinic ahead of schedule received telemedical treatment via telephone. Data collection to assess the patients’ and therapists’ satisfaction with and acceptance of the telemedical care was adjusted to the treatment setting.

**Results:**

Of 60 recruited patients, 57 could be included in the analysis. 51.6% of the patients and 52.3% of their therapists reported that the discussion of problems and needs worked just as well over the phone as in face-to-face consultations. In the subgroup of patients who were new to therapy due to being discharged from hospital early, acceptance was higher and telemedicine was rated as equally good in 87.5% of contacts. Both patients and therapists felt that telemedicine care during lockdown was an alternative for usual therapy in the outpatient clinic and that the option of telemedicine care should continue for the duration of the coronavirus pandemic.

**Discussion:**

The results show a clear trend towards satisfaction with and acceptance of telemedicine care in a heterogeneous group of unselected psychiatric patients. Although the number of patients is small, the results indicate that the mostly positive results of telemedicine concepts in research projects can probably be transferred to real healthcare settings.

**Conclusions:**

Telemedicine can be employed in healthcare for psychiatric patients either an alternative treatment option to maintain continuity of care or as a potential addition to regular care.

## Introduction

Since the onset of the COVID-19 pandemic in Germany in February 2020, there has been an increase in psychological distress in the general population in Germany. In a cross-sectional study with 15,704 German residents aged 18 years and older, participants reported significant increases in generalized anxiety, depression, psychological distress, and COVID-19-related anxiety due to the coronavirus pandemic [1].

Patients with mental disorders often suffer more from the resulting conditions and the consequences of the pandemic than persons without such a disease. In a study of 538 inpatients with mental disorders more than 50% showed a worsening of symptoms during the pandemic [2].

A meta-analysis by Neelam et al. showed that people with preexisting mental illness had significantly more psychiatric symptoms, anxiety symptoms, and depressive symptoms during the pandemic compared to healthy persons [3].

In March 2020, a general lockdown was imposed throughout Germany due to the coronavirus pandemic. One consequence was that hospitals were either no longer allowed to perform elective treatments or only to a limited extent. This restriction also affected patients with mental illnesses. Especially day-care and treatment by the outpatient clinics of the hospitals were discontinued. In many clinics, attempts were made to ensure continuity of care through telemedical support. Therapeutic treatment appointments were conducted by telephone or video conferencing [4]. A longitudinal observational study using data from the North Carolina Statewide Telepsychiatry Program found an association between the COVID-19 pandemic and increased demand for telepsychiatric consultations in the United States [5].

Telemedicine is already frequently used in psychiatry and psychotherapy for a broad range of psychiatric disorders, but so far mostly for selected patient groups and/or in the context of studies. The results of these studies are often positive. In the area of anxiety disorders, a meta-analysis of nine randomized controlled trials with a total of 1,837 participants showed that smartphone interventions resulted in a reduction of the total number of states of anxiety [6]. A systematic review and meta-analysis by Krzyzaniak et al. evaluated five small randomized controlled trials examining the effectiveness of telemedicine compared to face-to-face interventions for anxiety disorders. These authors found that the effectiveness of telemedicine interventions and face-to-face therapy was comparable [7]. Both a systematic review by Lim et al. of 6 studies and a randomized controlled trial (RCT) of patients with anxiety disorders and depression demonstrated that telemedicine care had a positive effect on the severity of psychiatric symptoms, particularly when the interventions occurred by telephone [8, 9]. An effective reduction of symptoms of depression and anxiety in women with postpartum depression who received telemedical treatment was also demonstrated in a systematic review and meta-analysis by Zhao et al., which included 9 RCTs with a total of 1,958 women [10]. Paalimäki-Paakki et al. found in their systematic review on the effectiveness of digital counseling interventions to improve anxiety, depression, and treatment adherence in chronically ill patients that digital, web-based counseling environments were comparable to or more effective than the usual counseling methods [11]. In a RCT by Dobkin et al., patients with comorbid depression in Parkinson’s disease received telephone-based cognitive-behavioral treatment (T-CBT), treatment as usual, or both. In this study, the intervention group with T-CBT showed significantly better scores for depression, anxiety, and quality of life compared to the control group with treatment as usual [12]. In a study by Schulze et al. a follow up of patients with schizophrenia or bipolar disorder was conducted via text messages and phone calls. Significantly better treatment adherence was found in the intervention group compared to the control group with usual care [13].

Acceptance of telemedicine care concepts is high among both practitioners [14, 15] and patients [16].

In an examination of 22 publications from different countries there was strong evidence for the feasibility and acceptability of telemedicine in mental health [17]. The results of a systematic literature review including fourteen studies showed that satisfaction with telemedicine for the treatment of depression is equal to t or greater than satisfaction with face-to-face treatment [18].

To our knowledge, there is little literature on the satisfaction with and acceptance of telemedicine in the outpatient psychiatric setting in Germany. A pilot study including patients with alcohol addiction by Haug et al. showed that telemedical interventions were well accepted [19].

However, so far there is little data available on the satisfaction of patients and therapists with telemedicine in psychiatric treatment during the COVID-19 pandemic. A high level of satisfaction with the use of telemedicine in psychiatry during the pandemic was demonstrated by a small study with 22 participants of a psychiatric day clinic in Skopje, Northern Macedonia. In this study, the overall satisfaction with telemedical care was high (80.2%) [20]. A systematic review by Dellazizzo et al. looking at the impact of COVID-19 on adults with neurocognitive disorders (NCD) showed that the pandemic resulted in exacerbations or relapses of neurocognitive symptoms and interrupted or reduced therapies for individuals with NCD. It was possible to alleviate these problems to some extent through the use of telemedical treatment [21].

The aim of this study was to examine the research question: “How feasible is telemedicine treatment for psychiatric patients of a psychiatric outpatient clinic and a day clinic of a university hospital in Germany to ensure the continuity of care during the first COVID-19 lockdown and how satisfied are the patients and therapists? “In addition, patient eligibility for telemedicine care was investigated.

## Methods

We followed the STROBE guidelines [22] for the description of the study as far as possible.

### Design

The study was designed as an observational study with a baseline assessment and at least one follow-up. The study was conducted in a regular healthcare setting.

### Patients

During the lockdown, from April 2020, almost all the patients of the psychiatric outpatient clinic and the day clinic of the university hospital Greifswald in the Northeast of Germany had to switch their treatment to telemedical care. This change affected patients receiving ongoing outpatient treatment, patients starting outpatient psychiatric care, as well as patients who had to be discharged from day-care treatment ahead of schedule. All patients were asked to participate in the study to evaluate the telemedical care, there were no inclusion or exclusion criteria. We included all patients from the clinic who were being treated there at the time of the lockdown.

### Telemedical intervention

Telemedical therapy could be performed by telephone or video conference. However, if necessary, face-to-face appointments were possible in compliance with strict rules of hygiene. In most cases, such face-to-face appointments took place outside in the park. The frequency, duration, and content of the therapeutic conversations depended on the individual needs of the patients. The telemedical appointments were carried out by physicians, therapists and nursing staff of the psychiatric outpatient clinic and the day clinic.

### Data assessment and procedures

Data was assessed in the context of the telemedicine treatment situation. In total, there were four different questionnaires. The questions were asked by the treating therapist, who also documented the answers. A standardized master data sheet was completed with each patient at the beginning of the telemedicine treatment, recording the patient’s name, gender, date of birth, main diagnoses and treatment phase (ongoing outpatient treatment, newly initiated outpatient treatment, treatment after early discharge from day care). For each contact, a contact questionnaire was completed which documented the date, time, duration of the appointment, contact type (scheduled or unscheduled), type of appointment (telephone, video conference or personal contact), and the main topics of the conversation. In addition, the standardized questionnaire “Brief Symptom Inventory” (BSI-18) was assessed at regular intervals (every 3–4 weeks if possible). The BSI-18 measures symptom severity and consists of a total of 18 questions with 6 questions each for depression, anxiety disorders, and somatoform disorders. The range for each item is from 0 (not at all) to 4 (very severe). The total BSI-18 score thus ranges from 0 to 72 points [23, 24].

At the beginning and at the end of the telemedical treatment and at every 4th appointment (at least once per quarter), a questionnaire was completed on the overall satisfaction with the telemedical treatment (“Better”, “Just as good”, “Worse”, “Sometimes better, sometimes worse”, “I don’t know”, “Not specified”), as well as satisfaction with the length of the conversations (“Exactly right”, “Too long”, “Too short”, “I don’t know”, “Not specified”), the frequency of the conversations (“Exactly right”, “Too often”, “Not often enough”, “I don’t know”, “Not specified”), and also whether telemedicine care is a good care option during the pandemic (“Yes”, “No”, “Neither yes nor no”, “I don’t know”, “Not specified”), the willingness to continue telemedicine care for the duration of the COVID-19 pandemic (“Yes”, “No”, “I don’t know”, “Not specified”), the question of returning to regular sessions instead of telemedicine support after the end of the pandemic (“I would like to get back to regular sessions as soon as possible”, “I would like to continue with the telephone support”, “I would like to combine the two options”, “I don’t know”, “Not specified”) and the impact of the pandemic on the patient’s overall well-being (“Positive impact”, “No impact”, “Negative impact”, “It depends on the situation”, “I don’t know”). In addition to the assessment of the patient’s perspective, the questionnaires also collected data on the therapist’s perspective. The therapist was asked whether telemedicine care was an appropriate form of care for the particular patient. All questionnaires were developed by the project team.

### Statistical analysis

The data were analyzed descriptively (means, standard deviations, ranges, frequencies, and percentages) on the basis of the complete dataset (intention to treat). We included all patients from the clinic who were being treated there at the time of the lockdown without inclusion or exclusion criteria. The sample therefore represents the typical patient group for this clinic, therefore, we didn’t control for confounding.

Stratified analyses were made for the question “How well are you able to discuss your problems and needs over the telephone with your therapist compared to a face-to-face conversation in the outpatient clinic?” because we expected different results regarding the research questions for these subgroups. Patients with missing data were excluded from the respective analyses.

For the questions “How do you feel about the length of the conversations?”, “How do you feel about the frequency of the conversations?”, “Is telemedicine care a good care option for you at Corona times as an alternative to the usual therapy in the outpatient clinic?” and “Would you like to continue telephone support for the duration of the Corona pandemic?” there were only very small differences between the subgroups, so for these questions we showed only the results for the total patient group.

## Results

The telemedical contacts took place between March 2020 and April 2021 during the COVID-19 pandemic. During this time, personal contacts were largely restricted at Greifswald University Hospital due to the contact restrictions in Germany. A total of 60 patients were included during this time period. Three of the patients had to be excluded from the analysis because of an incomplete medical history. This resulted in *N* = 57 patients who could be included in the analysis. Of these, 15 were male (26.3%). The age ranged from 20 to 78 years (mean: 44.2; standard deviation: 15.04 years). The majority of the patients (*n* = 42; 73.7%) were in an ongoing outpatient therapy at the time of the transition to telemedicine, 12.3% had been discharged early from day care, and 8.8% had started a new therapy. Moderate recurrent depressive disorder (43.9%) and posttraumatic stress disorder (26.3%) were the most frequent diagnoses (Table 1).

**Table 1:** Basic characteristics of the patient group receiving telemedicine treatment

	n	%
Full sample (patients)	57	100
Sex		
Female	39	68.4
Male	15	26.3
Diverse	1	1.8
Not Specified	2	3.5
Age		
20 – 29 years	10	17.5
30 – 39 years	17	29.8
40 – 49 years	7	12.3
50 – 59 years	12	21.1
60 + years	11	19.3
Minimum	20	
Maximum	78	
Median	46	
Mean	44.2	
Standard deviation	15.04	
Therapy stage		
Ongoing therapy	42	73.7
Newly started therapy	5	8.8
Early hospital discharge	7	12.3
Not specified	3	5.3
BSI (at baseline)		
Minimum	0	
Maximum	52	
Median	17	
Mean	18.7	
Standard deviation	13.69	
Most frequent diagnoses		
F33.1	Recurrent depressive disorder, currently moderate episode	43.9%
F43.1	Posttraumatic stress disorder	26.3%
F33.2	Recurrent depressive disorder, currently severe episode without psychotic symptoms	15.8%
F61	Combined and other personality disorders	10.5%
F45.41	Chronic pain disorder with somatic and psychological factors	8.8%

The BSI-18 score ranged from 0 to 52 at the start of the telemedicine treatment (median: 17; mean: 18.7; standard deviation: 13.69).

Of a total of 280 contacts, the therapist was a psychiatrist 24 times (8.57%), a psychologist 166 times (59.29%), and a nurse 86 times (30.71%). In 4 contacts there was no specification (1.43%).

A total of 280 treatment contacts between patients and treatment providers were documented. The number of contacts ranged from one to 12 per patient (median: 2; mean: 2.2; standard deviation: 1.66). Contacts occurred over a time period of one to 12 months (median: 2; mean: 3.16; standard deviation: 2.72). Most of the patient contacts were scheduled (*n* = 264; 94.3%). *n* = 202 contacts took place by telephone (72.1%), the call duration ranged from one minute to four hours (mean duration: 49 min 29 s, standard deviation: 21 min 1 s).

The main conversation topics were “coping with/managing the disease, symptoms, and limitations” (58.2%), “creating/maintaining daily structure” (36.1%), “managing daily activities” (32.1%), and “dealing with changes/restrictions due to the COVID-19 pandemic” (29.3%). The contact characteristics are shown in Table 2.

**Table 2:** Contact characteristics of the patient group receiving telemedicine treatment

	n	%
Full sample (contacts)	280	100
Contact type		
Planned contact	264	94.3
Unplanned contact	13	4.6
Not specified	3	1.1
Contact media		
Contact by phone	202	72.1
Personal contact	73	26.1
Not specified	5	1.8
Therapist type		
Psychologist	166	59.3
Nursing staff	86	30.7
Psychiatrist	24	8.6
Not specified	4	1.4
Number of contacts		
Minimum	1	
Maximum	12	
Median	2	
Mean	2.21	
Talk time		
Minimum	1 minute	
Maximum	4 hours	
Mean	49 min 29 s	

The questionnaire on satisfaction and acceptance was completed on average 2.2 times per patient (standard deviation: 1.62). The question, “How well can you discuss your problems and needs over the telephone with your therapist compared to a face-to-face conversation in the outpatient clinic?” was answered with “just as well” in most cases (51.6% of the patient contacts (*n* = 66) and 52.3% of therapist contacts (*n* = 67)). For patient contacts “Better” was the documented response in 3.9% of cases (*n* = 5) and for therapist contacts this was the response in about 3.1% of cases (*n* = 4).

**Fig. 1:**
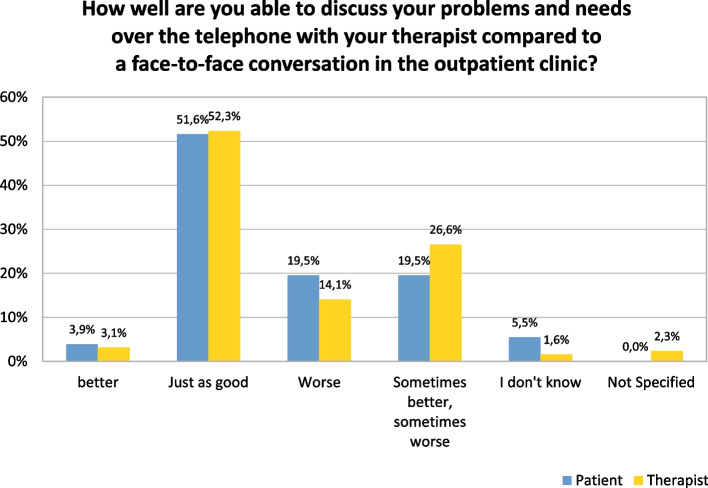
Satisfaction of patients and therapists with telemedicine treatment

With respect to this question there was complete agreement between the perception of the patient and that of the therapist in 51.6% of cases (*n* = 66). Mostly, both the patient and the therapist classified the possibility of discussing problems by means of telemedicine contacts as “just as good” as face-to-face therapy (*n* = 45; 68.2%). Discussion of problems by telemedicine was seen as “worse” by 19.5% of patient contacts (*n* = 25) and 14% of therapist contacts (*n* = 18). In eight contacts, the therapist and the patient both saw telemedicine contacts as “worse” than face-to-face contacts. The main reason for thinking that “face-to-face contact” was better, was that facial expressions and gestures were missing in telephone conversations (Fig. 1 and Table 3).

**Table 3:** Satisfaction and evaluation of telemedicine care of patients and therapists with telemedicine treatment

	n	%				
Full sample (questionaires)	128	100				
How well are you able to discuss your problems and needs over the telephone with your therapist compared to a face-to-face conversation in the outpatient clinic?						
	total patient		total therapist			
	n	%	n	%		
Better	5	3.9	4	3.1		
Just as good	66	51.6	67	52.3		
Worse	25	19.5	18	14.1		
Sometimes better, sometimes worse	25	19.5	34	26.6		
I don’t know	7	5.5	2	1.6		
Not specified	0	0	3	2.3		
	male patient		female patient			
	n	%	n	%		
Better	1	2.9	3	3.4		
Just as good	17	50.0	47	54.0		
Worse	7	20.6	16	18.4		
Sometimes better, sometimes worse	7	20.6	17	19.5		
I don’t know	2	5.9	4	4.6		
	Ongoing therapy		new started therapy		early hospital discharge	
	n	%	n	%	n	%
Better	1	1.1	1	14.3	1	4.2
Just as good	40	44.4	4	57.1	21	87.5
Worse	22	24.4	0	0	1	4.2
Sometimes better, sometimes worse	21	23.3	2	28.6	1	4.2
I don’t know	6	6.7	0	0	0	0
	20–29 years (*n* = 10)		30–39 years (*n* = 17)		40–49 years (*n* = 7)	
	n	%	n	%	n	%
Better	0	0	1	2.2	1	7.7
Just as good	8	38.1	22	47.8	6	46.2
Worse	4	19.0	11	23.9	5	38.5
Sometimes better, sometimes worse	7	33.3	9	19.6	1	7.7
I don’t know	2	9.5	3	6.5	0	0
	50–59 years (*n* = 12)		60 + (*n* = 11)			
	n	%	n	%		
Better	3	10.7	0	0		
Just as good	18	64.3	12	60.0		
Worse	2	7.1	3	15.0		
Sometimes better, sometimes worse	5	17.9	3	15.0		
I don’t know	0	0	2	10		
How do you feel about the length of the conversations?						
	total patient		total therapist			
	n	%	n	%		
Exactly right	105	82.0	118	92.2		
Too long	4	3.1	4	3.1		
Too short	5	3.9	2	1.6		
I don’t know	14	10.9	2	1.6		
Not specified	0	0	2	1.6		
How do you feel about the frequency of the conversations?						
	total patient		total therapist			
	n	%	n	%		
Exactly right	101	78.9	115	89.8		
Too often	1	0.8	1	0.8		
Not often enough	13	10.2	6	4.7		
I don’t know	13	10.2	3	2.3		
Not specified	0	0	3	2.3		
Is telemedicine care a good care option for you at Corona times as an alternative to the usual therapy in the outpatient clinic?						
	total patient		total therapist			
	n	%	n	%		
Yes	109	85.2	114	89.1		
No	9	7.0	4	3.1		
Neither yes nor no	3	2.3	7	5.5		
I don’t know	6	4.7	0	0		
Not specified	1	0.8	3	2.3		
Would you like to continue telephone support for the duration of the Corona pandemic?						
	total patient		total therapist			
	n	%	n	%		
Yes	101	78.9	103	80.5		
No	22	17.2	19	14.8		
I don’t know	4	3.1	2	1.6		
Not specified	1	0.8	4	3.1		
Patients only: Would you like to return to regular sessions after the Corona pandemic ends instead of phone support?						
					n	%
I would like to get back to regular sessions as soon as possible					72	56.3
I would like to continue with the telephone support					1	0.8
I would like to combine the two options					51	39.8
I don’t know					3	2.3
Not specified					1	0.8
Patients only: What impact does the Corona pandemic have on your well-being?						
	n	%				
Positive impact	11	8.6				
No impact	19	14.8				
Negative impact	38	29.7				
It depends on the situation	59	46.1				
I don’t know	1	0.8				

Differences in the evaluation of telemedicine were seen with respect to the stages of therapy. Patients in an ongoing therapy saw the telemedical contacts less positively than new patients; they were described as “just as good” in 44.4% of the contacts (*n* = 40), and 24.4% of the cases (*n* = 22) even perceived them as “worse”. Of the patients who had started a new therapy, 57.1% saw the contacts as “just as good” (*n* = 4), and none of the 5 patients felt that telemedical contacts were “worse”.

The acceptance questionnaire was completed 24 times by patients who were discharged from day care ahead of schedule (*n* = 7 patients). For the most part (87.5%), these patients saw the telephone conversation as “just as good” as face-to-face conversations (*n* = 21) (Table 3).

**Fig. 2:**
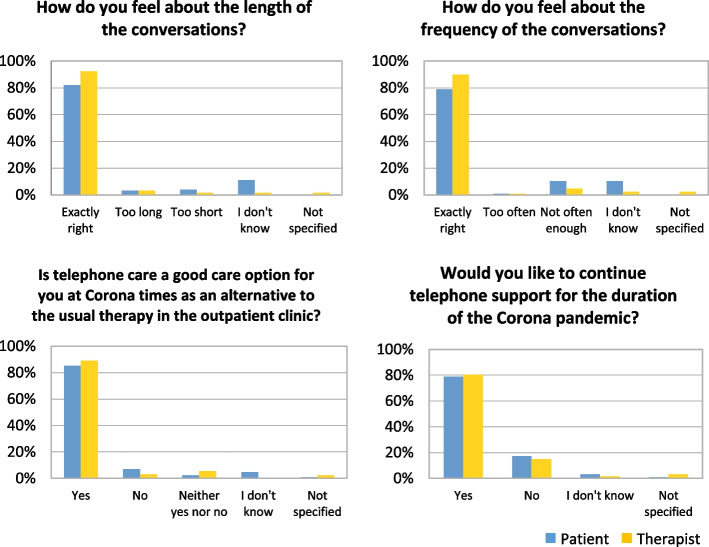
Evaluation of telemedicine care of patients and therapists with telemedicine treatment

The optional text box “Which topics can you not discuss? Which topics particularly well?” was completed in 45 of 128 contacts (35.2%). The most frequent comment was that facial expressions and gestures were lacking during telephone contact. On the other hand, among those discharged ahead of schedule, it also occurred frequently (12 times) that no restrictions were perceived and that everything could be discussed (see Table 3 and Fig. 2).

In most cases, both patients and therapists answered “yes” to the question as to whether telemedicine care was a good alternative to the usual therapy during the COVID-19 lockdown (patients: 85.2%, *n* = 109, therapists: 89.1%, *n* = 114). In most cases respondents approved of the continuation of telemedicine care for the duration of the pandemic (patients: 78.9%, *n* = 101, therapists: 80.5%, *n* = 103) (See Table 3 and Fig. 2).

The option of care using solely telemedicine after the end of the pandemic was met with approval in only one case; rather, patients wanted to combine telemedicine and face-to-face therapy (39.8%, *n* = 51) or return to regular face-to-face sessions (56.3%, *n* = 72).

In terms of the influence of the coronavirus pandemic on patients’ well-being the main responses were “it depends on the situation” (46.1%, *n* = 59), or as “negative” (29.7%, *n* = 38).

The question “Is telemedicine care appropriate for this patient?” was completed 128 times, exclusively by the therapists. In most cases (63.3%, *n* = 81) this was answered with “yes”.

## Discussion

In our evaluation, the first question “How well are you able to discuss your problems and needs over the telephone with your therapist compared to a face-to-face conversation in the outpatient clinic?” was an important focus of our evaluation. Therefore, stratified analyses were conducted for this question.

In most cases, it was stated that discussing problems and needs on the phone worked just as well as in face-to-face meetings, with only minor differences between the genders and age groups. Patients who were new to therapy due to early hospital discharge were even more accepting. Both patients and therapists perceived telemedicine care during the lockdown as an alternative to usual therapy in the outpatient clinic and in most cases were in favor of continuing telemedicine care for the duration of the pandemic.

In the psychiatric outpatient department where this study was conducted, there was already considerable experience with telemedical care for patients with depression, anxiety disorders, schizophrenia and bipolar disorders, initially in the context of research projects and subsequently also in regular care. However, telemedicine was usually carried out after treatment in the day clinic and suitable patients were specifically selected.

Due to the general lockdown in Germany from March 2020, it was necessary to switch from face-to-face therapy sessions to telemedical care for all patients at short notice. Whether the patients accepted this and how the therapists assessed the telemedical treatment was analyzed in this observational cohort study.

The largest difference to the previous telemedicine treatment was the selection of the patients. The patient group here was not pre-selected, but rather a “normal” patient group with a large variation in diagnoses, disease severity and phase of treatment. The results showed a clear trend toward acceptance of and satisfaction with telemedicine care. More than half of the patients and a similar proportion of the therapists considered telephone treatment to be “just as good” as face-to-face treatment.

With respect to the acceptance of and satisfaction with the telemedicine therapy, there are no major differences between genders or age groups. In all groups, the prevailing view was that problems and needs could be discussed “just as well” on the phone as in a personal conversation in the outpatient clinic.

One possible explanation as to why patients in an ongoing therapy were somewhat less accepting and satisfied could be the fact that the individual face-to-face conversations that they were accustomed to suddenly stopped. Patients with a newly initiated therapy felt that telemedicine was “just as good” more often than average. This observation could be explained by the fact that they had not yet had any personal contact with the therapist beforehand, which would have served as a comparison.

The question “Would you like to return to regular sessions after the coronavirus pandemic ends instead of phone support?” also showed that telemedicine has its place in psychiatric care and can be used as a blended treatment. Although most patients would like to return to regular therapy, 39.8% of the cases desired a combination of both therapy options.

Our study shows conformity with study results from other countries. A review of 196 articles by Abraham et al. on the use of telemedicine care during the COVID-19 pandemic, which primarily examined the scope and areas of telemental health rather than its acceptance, found that telemedicine use increased substantially during the pandemic. Moreover, it was effective and safe and would continue to be used for the foreseeable future [25].

In a quantitative cross-sectional study conducted in Austria and Germany in 2020, 190 psychotherapists were surveyed regarding the comparability of psychotherapy via telephone with face-to-face contact. The results showed that there was a positive correlation between both the number of patients treated via telephone and the therapist’s experience with telemedicine psychotherapy and the therapist’s perceived comparability of the different types of therapy [26].

A systematic review by Siegel et al. published during the COVID-19 pandemic summarized the obstacles to telemedicine as technological difficulties; issues of security, privacy, and confidentiality; therapeutic implementation; and the physician–patient relationship [27].

However, with respect to satisfaction with the therapy, the results are in line with the results from studies before the pandemic. Both therapists [14, 15] and patients [16] show a high level of acceptance of telemedicine. However, no videoconferencing was performed during our observation period, as there was little experience with this technology during the first wave of COVID-19.

Our study has some limitations. Due to the small number of participants in our study we could only conduct descriptive statistics. Analytical statistics could not be carried out. Due to the broad range of diagnoses and the fact that patients with more than one diagnosis were included, t was not possible to analyze satisfaction with medical care with respect to specific diagnoses.

We analyzed satisfaction with telemedicine on a case basis from the perspective of both the patient and the therapist. It is likely that questionnaires completed by the same patients and dyads are not independent of each other. However, the dyads often changed during the observation phase of the study although the observation period was relatively short. This short duration was mainly related to the gradual relaxation of contact restrictions from March 2021, and the face-to-face appointments that were possible again from then on.

We cannot exclude a selection bias because we do not know whether patients broke off contact with the clinic during the lockdown thus not receiving any treatment.

Since telemedicine care was the regular form of healthcare during the COVID-19 lockdown, a controlled or randomized trial with a control group was not possible. This might be a limitation for the interpretation and reliability of the results. However, the observation of the regular healthcare setting is also a strength. The analysis is a representation of real-world care, as all patients in the psychiatric outpatient clinic and the day hospital had to be transferred to telemedicine therapy, and thus the evaluation represents the broad spectrum of psychiatric patients in routine outpatient care.

## Conclusion

Telemedicine provides an alternative modality in the care of psychiatric patients when circumstances prohibit face-to-face encounters, such as in a pandemic situation. Hence telemedicine can help ensure the continuity of care.

In addition, our data supports the notion that telemedicine provides a potential extension of therapy modalities for patients in outpatient psychiatric care, also outside of the pandemic situation.

## Data Availability

The data for this analysis come from the care setting and, in part, directly from the medical records of the participating patients. Patients have consented to us using this data for analysis, however not to making the original data publicly available. The number of patients is small, all patients come from the same region, the data collection period is limited, and all patients have psychiatric diagnoses. An identification of the patients on the basis of the original data can therefore not be ruled out. Prof. Dr. Neeltje van den Berg can be contacted concerning requests for data from this study.

## References

[1] Bäuerle A, Teufel M, Musche V, Weismüller B, Kohler H, Hetkamp M (2020). Increased generalized anxiety, depression and distress during the COVID-19 pandemic: a cross-sectional study in Germany. J Public Health (Oxf).

[2] Favreau M, Hillert A, Osen B, Gärtner T, Hunatschek S, Riese M (2021). Psychological consequences and differential impact of the COVID-19 pandemic in patients with mental disorders. Psychiatry Res..

[3] Neelam K, Duddu V, Anyim N, Neelam J, Lewis S (2021). Pandemics and pre-existing mental illness: a systematic review and meta-analysis. Brain Behav Immun Health.

[4] Colbert GB, Venegas-Vera AV, Lerma EV (2020). Utility of telemedicine in the COVID-19 era. Rev Cardiovasc Med.

[5] Xue Y, Saeed SA, Liang H, Jones K, Muppavarapu KS (2022). Investigating the impact of Covid-19 on telepsychiatry use across sex and race: a study of north Carolina emergency departments. Telemed J E Health.

[6] Firth J, Torous J, Nicholas J, Carney R, Rosenbaum S, Sarris J (2017). Can smartphone mental health interventions reduce symptoms of anxiety? A meta-analysis of randomized controlled trials. J Affect Disord.

[7] Krzyzaniak N, Greenwood H, Scott AM, Peiris R, Cardona M, Clark J, Glasziou P. The effectiveness of telehealth versus face-to face interventions for anxiety disorders: a systematic review and meta-analysis. J Telemed Telecare. 2021:1357633X211053738. 10.1177/1357633X211053738. 10.1177/1357633X211053738 34860613

[8] Lim CT, Rosenfeld LC, Nissen NJ, Wang PS, Patel NC, Powers BW, Huang H (2022). Remote care management for older adult populations with elevated prevalence of depression or anxiety and comorbid chronic medical illness: a systematic review. J Acad Consult Liaison Psychiatry.

[9] van den Berg N, Grabe H-J, Baumeister SE, Freyberger HJ, Hoffmann W (2015). A telephone- and text message-based telemedicine concept for patients with mental health disorders: results of a randomized controlled trial. Psychother Psychosom.

[10] Zhao L, Chen J, Lan L, Deng N, Liao Y, Yue L (2021). Effectiveness of telehealth interventions for women with postpartum depression: systematic review and meta-analysis. JMIR Mhealth Uhealth.

[11] Paalimäki-Paakki K, Virtanen M, Henner A, Nieminen MT, Kääriäinen M (2022). Effectiveness of digital counseling environments on anxiety, depression, and adherence to treatment among patients who are chronically ill: systematic review. J Med Internet Res.

[12] Dobkin RD, Mann SL, Gara MA, Interian A, Rodriguez KM, Menza M (2020). Telephone-based cognitive behavioral therapy for depression in Parkinson disease: a randomized controlled trial. Neurology.

[13] Schulze LN, Stentzel U, Leipert J, Schulte J, Langosch J, Freyberger HJ, et al. Improving Medication Adherence With Telemedicine for Adults With Severe Mental Illness. Psychiatr Serv. 2019;70:225–8. 10.1176/appi.ps.201800286. 10.1176/appi.ps.201800286 30651059

[14] Peine A, Paffenholz P, Martin L, Dohmen S, Marx G, Loosen SH (2020). Telemedicine in Germany during the COVID-19 pandemic: multi-professional national survey. J Med Internet Res.

[15] Montoya MI, Kogan CS, Rebello TJ, Sadowska K, Garcia-Pacheco JA, Khoury B (2022). An international survey examining the impact of the COVID-19 pandemic on telehealth use among mental health professionals. J Psychiatr Res.

[16] Chipps J, Brysiewicz P, Mars M (2012). Effectiveness and feasibility of telepsychiatry in resource constrained environments? A systematic review of the evidence. Afr J Psych.

[17] Bashshur RL, Shannon GW, Bashshur N, Yellowlees PM (2016). The empirical evidence for telemedicine interventions in mental disorders. Telemed J E Health.

[18] Guaiana G, Mastrangelo J, Hendrikx S, Barbui C (2021). A systematic review of the use of telepsychiatry in depression. Community Ment Health J.

[19] Haug S, Lucht MJ, John U, Meyer C, Schaub MP (2015). A pilot study on the feasibility and acceptability of a text message-based aftercare treatment programme among alcohol outpatients. Alcohol Alcohol.

[20] Haxhihamza K, Arsova S, Bajraktarov S, Kalpak G, Stefanovski B, Novotni A, Milutinovic M (2021). Patient satisfaction with use of telemedicine in University clinic of psychiatry: Skopje, North Macedonia during COVID-19 pandemic. Telemed J E Health.

[21] Dellazizzo L, Léveillé N, Landry C, Dumais A (2021). Systematic review on the mental health and treatment impacts of COVID-19 on neurocognitive disorders. J Pers Med.

[22] von Elm E, Altman DG, Egger M, Pocock SJ, Gøtzsche PC, Vandenbroucke JP (2007). The Strengthening the Reporting of Observational Studies in Epidemiology (STROBE) statement: guidelines for reporting observational studies. Lancet.

[23] Derogatis LR, Melisaratos N (1983). The brief symptom inventory: an introductory report. Psychol Med.

[24] Spitzer C, Hammer S, Löwe B, Grabe HJ, Barnow S, Rose M (2011). Die Kurzform des Brief Symptom Inventory (BSI -18): erste Befunde zu den psychometrischen Kennwerten der deutschen Version. [The short version of the Brief Symptom Inventory (BSI -18): preliminary psychometric properties of the German translation]. Fortschr Neurol Psychiatr.

[25] Abraham A, Jithesh A, Doraiswamy S, Al-Khawaga N, Mamtani R, Cheema S (2021). Telemental health use in the COVID-19 pandemic: a scoping review and evidence gap mapping. Front Psychiatry.

[26] Korecka N, Rabenstein R, Pieh C, Stippl P, Barke A, Doering B (2020). Psychotherapy by telephone or internet in Austria and Germany which CBT psychotherapists rate it more comparable to face-to-face psychotherapy in personal contact and have more positive actual experiences compared to previous expectations?. Int J Environ Res Public Health.

[27] Siegel A, Zuo Y, Moghaddamcharkari N, McIntyre RS, Rosenblat JD (2021). Barriers, benefits and interventions for improving the delivery of telemental health services during the coronavirus disease 2019 pandemic: a systematic review. Curr Opin Psychiatry.

